# Symptomatology Correlations Between the Diaphragm and Irritable Bowel Syndrome

**DOI:** 10.7759/cureus.3036

**Published:** 2018-07-23

**Authors:** Bruno Bordoni, Bruno Morabito

**Affiliations:** 1 Cardiology, Foundation Don Carlo Gnocchi (IRCCS)/Institute of Hospitalization and Care, Milano, ITA; 2 Osteopathy, School of Osteopathic Centre for Research and Studies, Rome, ITA

**Keywords:** irritable bowel syndrome, pain, diaphragm, low back pain, chronic headache

## Abstract

Irritable bowel syndrome (IBS) is one of the most debilitating and common gastrointestinal disorders; nevertheless, its pathophysiology is still unclear. It affects 11% of the world's population, and is characterized by alternating periods of pain and/or motility disorders with periods of remission and without any evidence of any structural and functional organic variation. It has been recently proposed that an altered contractile ability of the diaphragm muscle might adversely influence intestinal motility. The text reviews the diaphragm's functions, anatomy, and neurological links in correlation with the presence of chronic symptoms associated to IBS, like chronic low back pain, chronic pelvic pain, chronic headache, and temporomandibular joint dysfunction, vagus nerve inflammation, and depression and anxiety. The interplay between an individual's breath dynamic and intestinal behaviour is still an unaddressed point in the physiopathology of IBS, and the paucity of scientific studies should recommend further research to better understand the importance of breathing in this syndrome.

## Introduction and background

Irritable bowel syndrome (IBS) is the most commonly diagnosed gastrointestinal disorder. The patient complains of abdominal pain or discomfort and altered bowel habits in the absence of concomitant organic diseases that could explain symptoms [1]. IBS can be divided into subgroups, depending on the prevailing symptoms (based on the scale of Rome III) and stool consistency (based on the Bristol Stool Form Scale): diarrhea or IBS-D; with constipation or IBS-C; alternating constipation and diarrhea, or IBS-M; untyped IBS or IBS-U (insufficient stool abnormalities to be IBS-C, D or M) [1-2]. Patients may have variations in their symptoms, with periods of remission; it is not easy to correctly classify patients with IBS [1-2]. Anyway, to diagnose IBS, symptoms have to persist for at least three months, with abdominal discomfort presenting 3-4 times per month, with constant feedback for 1-2 years; after 10 years, 50-70% of patients still present symptoms [3]. IBS currently refers to a disease of unknown aetiology.

The most recent hypotheses

The most recent hypotheses concern the presence of intestinal dysbiosis, which may affect both the large and small bowel tract. Many factors participate in the alteration of intestinal microbiota, such as a recent history of acute intestinal infections or reduction of intestinal motility [4]. Another hypothesis is that the presence of small intestinal bacterial overgrowth (SIBO), which causes a non-physiological post-prandial fermentation with elevated gas production, results in rapid gastric (painful) distension. SIBO is probably responsible for chronic and sub-clinical inflammation of the intestinal mucosa and could also explain an elevation in a mucosal specific inflammatory protein, calprotectin, which may be found in these patients [5]. Other theories deal with the role of serotonin, a neurotransmitter involved in multiple intestinal functions, and able to influence motility, immune response, and visceral sensitivity [6]. Intestinal receptors and serotonin uptake may be possibly involved in its pathogenesis, especially in IBS-D and IBS-C, but additional data are certainly needed to clarify serotonin’s role in IBS aetiology [5]. The central nervous system may be also involved in the pathogenetic mechanism leading to IBS. The brain-gut axis is considered as the bi-directional relationship between the enteric nervous system and the central nervous system, including the hypothalamus-pituitary-adrenal gland axis [5]. When this mechanism is altered, some visceral allodynia could appear, due to the increased release of cortisol and pro-inflammatory cytokines, increasing symptoms [4]. Stress generates an endocrine response (corticotropin-releasing factor (CRF), activating in turn the hypothalamic-pituitary-adrenal axis (HPA). CRF is secreted by the paraventricular nucleus of the hypothalamus, the release of which is regulated by the amygdala (the limbic system). The CRF receptors (CRF1 and CRF2) are found on enteric neurons and at the mucosal layer. The constant presence of stress could cause a pathophysiological change in the intestine, altering its motility (mobility, relaxation, transit) through the activation of these receptors [4]. This fibromyalgia-like phenotype is often diagnosed in patients affected by IBS and it is characterized by chronic fatigue, chronic back pain, chronic pelvic pain, chronic headache, and temporomandibular joint dysfunction, at a rate almost twice the general population [3]. Some authors call such symptoms "functional somatic syndromes" and have therefore proposed to treat these patients with selective serotonin receptive inhibitors (SSRI) or tricyclic antidepressants. In addition, more than half of the patients reported symptoms of anxiety and depression; the risk of developing dementia in subjects (after 50 years) is increased [3-7]. The interest in the role of the diaphragm muscle in the context of functional intestinal disorders is justified by its autonomic dependent function and because it is the only striated muscle whose movement affects the small and large bowel. The purpose of this article is to review the importance of the diaphragm muscle in the context of IBS symptoms, since there are many studies that show that an alteration of its contractile ability might have a negative impact on the pathology.

## Review

Diaphragm

The diaphragm muscle is innervated by the phrenic nerve (C3-C5) and the vagus nerve (cranial nerve X); the first receives pulses from groups of medullary neurons of the pre-Bötzinger complex and from neurons of the parafacial retrotrapezoid complex, connected in turn with the retroambiguus nucleus of medulla, although the underlying mechanisms of these connections are not completely clear. The vagus nerve is part of the parasympathetic autonomic system originating from the ambiguus nucleus of the medulla [8-9].

Symptomatology correlations

The abdominal muscles, the rectus, and the oblique and transverse muscles not only play a biomechanical role in relation with the spine, but also in relation to intra-abdominal content, changing their electrical activity according to intestinal pressure [10]. In patients affected by IBS, this accommodation mechanism of the abdominal muscles does not work properly, and an electromyographic alteration can be observed in postprandial periods. The patients experience a paradoxical effect: a contraction of the diaphragm muscle and a relaxation of the upper portion of the abdominal wall, while in healthy subjects, concomitant relaxation of the diaphragm muscle and activation of the rectus abdominis (the upper part) and external oblique usually happen. This phenomenon causes abdominal swelling, and probably dyspepsia [10]. This lack of motor coordination between the diaphragm and the abdominal wall is called "abdomino-phrenic dyssynergia" [11]. During respiration, the muscles of the abdominal wall and the diaphragm muscle are controlled by the same centers of the retroambiguus phrenic nucleus in the medulla, in an electric combination that allows a perfect synergic contraction during inspiration and expiration [8]. A dysfunction of the diaphragm muscle may alter this functional synergy and cause an alteration in the motor scheme. This event could lead to swelling and dyspepsia in these patients.

Abnormalities in the pelvic area and in the upper airways

The phrenic centers in the medulla control the pelvic floor muscles and the tongue during respiration [8-9]. During inspiration, the tongue protrudes (to open the upper airway), while the pelvic floor undergoes a downward movement; during expiration, the opposite happens. If this mechanism is altered for a dysfunction of the diaphragm, abnormalities in the pelvic area (chronic pelvic pain) and in upper airways can be observed [8-9]. Patients with IBS are affected by chronic pelvic pain in 35%-80% of cases worldwide; in addition, collapsibility of the upper airways during sleeping is observed in 15%-44% of cases, as reported in a pilot study [12-13].

Low back pain

Low back pain can be also related to IBS, although its aetiology is not completely clear [14]. The diaphragm plays a fundamental role in defining posture, its maintenance, and body position changes. The dysfunction of the diaphragm is one of the recognized causes of low back and sacroiliac joint pain. People with low back pain often experience early fatigue of the diaphragm muscle, altered and reduced excursion during respiration, and inadequate proprioceptive activation [15]. The diaphragm dynamically stabilizes the lumbar spine. Lowering during inhalation together with the latest coasts, it stabilizes abdominal pressure, with a deeper movement in its ventral portion than in the dorsal area. In people with chronic lower back pain, the diaphragm remains higher and more flattened, with inadequate movement of the ventral portion. When the legs move, the diaphragm stabilizes the spine allowing the required movement; this does not happen correctly in people suffering from chronic pain. The coasts do not drop and do not allow the diaphragm to have fixity; there is a minor drop in the diaphragmatic cupola with a reduced ability to manage the intra-abdominal pressure, consequently causing lumbar spine instability [15-17]. Since a dysfunction of the electrical activation of the diaphragm has been demonstrated in patients with IBS, we can assume that one of the causes leading to low back pain in this population may be altered activation of the main respiratory muscle.

Temporomandibular joint dysfunction

Another somatic disorder affecting IBS patients is temporomandibular joint dysfunction with pain [3]. The functional mechanisms connecting the diaphragm and tongue during normal respiration are well known, as well as their neurological connections, either central (medulla) and peripheral (anastomosis of the phrenic nerve and hypoglossal nerve at the level of the ansa cervicalis) [8]. Upper airways abnormalities, together with difficult coordination of tongue activity, can alter kinematics of the temporomandibular joint [18]. This lets us presume even a close relationship between these symptoms, diaphragm dysfunction, and IBS.

Chronic headaches

To better understand the presence of chronic headaches in IBS and its relations with diaphragmatic functions, the activity of the thoracolumbar fascia should be considered. The latter is a multi-layered fascial and muscular structure that covers the entire abdominal muscles and the myofascial area between the sacrum and the cervical area, from the surface to depth; the diaphragm is directly connected with the thoracolumbar fascia [8]. All fascial layers are inseparable; they move simultaneously, relate to one another, and influence each other's activity [19]. The thoracolumbar fascia properly conveys the tensions generated by movements and breathing along the spine, creating a synergy between the diaphragm and daily activities [15]. The sub-occipital muscles (a couple of recti and a couple of oblique muscles) are part of the thoracolumbar fascia and are innervated by the first cervical branches [8]. The recti muscles and the superior oblique sub-occipital muscle have a myodural bridge to the dura mater [20]. In the connective tissue of the dura, near the arterial and venous vessels, there are some peculiar receptors with functions similar to Ruffini receptors and which have highly mechano-sensitive and chemical-sensitive responses, and nociceptors [21]. Abnormal pressures recorded by the dura mater, caused by an abnormal tension derived from the myodural bridges of the sub-occipital muscles, may be the cause of the chronic headaches [22]. Diaphragmatic dysfunctions affect the whole system, including sub-occipital muscles [8-9]. The incorrect functioning of the diaphragm could cause an increase in work for the accessory respiratory muscles. We can assume that the overloading of the sternocleidomastoideus and scalene muscles could be another cause of headaches that result from the primary dysfunction of the diaphragm. A correlation between chronic headaches and the altered function of the diaphragm muscle can be assumed.

Gastroesophageal reflux

Gastroesophageal reflux (GERD) is another symptom frequently observed in patients with IBS (around 40%) [23]. The diaphragmatic crura play a role as extrinsic sphincter in the region of the gastroesophageal junction to protect the esophagus from gastric reflux; a crural dysfunction can cause GERD [24]. Therefore, a relationship between IBS and GERD can be reliably established in case of diaphragmatic dysfunction.

Anxiety, depression, and perceived pain

Psychiatric disorders such as depression and anxiety are quite common in patients affected by IBS; they have been observed in 46% and 34% of cases respectively [5]. These behavioural changes may be due to disease-related stress, since the severity of the emotional disorder is often correlated with the severity of the symptoms of IBS [5]. Chronic altered emotional states can cause changes in neural and structural patterns of emotional perception and thus increase pain perception [25]. Another hypothesis deals with the persistent solicitation of visceral nociceptors in response to mechanical stress (inappropriate or exaggerated colic distension, inflammation, and ischemia), potentially causing visceral hyperalgesia or allodynia. This peripheral perception may induce changes in the central regulation, establishing a vicious circle between visceral and emotional information, and pain perception [25]. The visceral afferents, interoceptive as well as nociceptive (small caliber A delta and C fibers) reach lamina I and II of the spinal cord, and are then projected to supraspinal centers, such as the posterior ventromedial nucleus of the thalamus and the limbic area [26]. Another reason to study the presence of psychiatric disorders in this patient population is to research its correlation with dysfunction of the diaphragm muscle and the relationship of this dysfunction with emotions and pain perception. The perception of pain is reduced if patients hold their breath after a deep breath, when the diaphragm is lowered [27]. This observation lets us presume the potential role of baroreceptors.

During inspiratory apnea, systolic pressure increases with a decrease in cardiac frequency. When the baroreceptors located in the carotid body and in the aortic arch area, in the adventitia of the vessels, are stimulated by the cardiac cycle, in particular during the systolic phase, the nociceptive stimulus is attenuated by the activation of baroreceptors [27]. The baroreceptors’ activity also affects muscle tone, as it decreases the activity of the sympathetic nervous system, reducing the contractile state. The baroreceptors are activated if the vessels are stretched by blood passage; afferent fibers conduct pulses to the nucleus of the solitary tract (NTS), which regulates the activation of the vagal system and sympathetic inhibition at the spinal level in the region of the nucleus ambiguus, dorsal motor nucleus, and rostral ventrolateral area of the medulla. The baroreceptorial afferents influence different areas of the central nervous system, with a generalized inhibitory effect. The NTS is interconnected with the reticular formation; the information will be then transmitted to the anterior (limbic area), latero-medial and prefrontal part of the insula and to the anterior cingulate cortex; even the thalamus, hypothalamus, and periaqueductal grey area receive baroreceptorial pulses from NTS [27]. The diaphragm with its movements changes the body pressure, as it facilitates venous and lymphatic return [8]. This pressure modulation influences blood re-distribution. This action may affect the response of baroreceptors and thus pain perception, but there are no scientific studies to support this statement yet. We can presume that the baroreceptors will not be adequately stimulated, in case of an alteration of the diaphragm motility; this would lead to an increased sensitivity to pain. A lower sensitivity of the baroreceptors and a higher pain perception have been demonstrated in IBS patients [28]. Chronic pain can alter the subject's emotional state [27]. We can speculate that the dysfunction of the diaphragm could be one of the causes of this psychiatric condition and high pain perception.

The diaphragm also influences emotions directly. The interaction between breathing and emotions reflects a complex interaction between the brainstem and some brain areas such as the limbic area and the cortex. The amygdala, which is part of the limbic system, and some respiratory areas located in the medulla, are connected to each other; thus, the amygdala is usually considered as the most important area in the interaction between breathing and emotional state. The amygdala is divided into three areas (basolateral, cortical and central); the basolateral amygdala conducts pulses to the central area, which is directly and indirectly connected to the hypothalamus and to the brainstem [27]. The amygdala is stimulated by dopamine production from the tegmental area of the midbrain; a recent study on animal models demonstrated the role of dopamine in the amygdala in the management of the emotional breath [29]. The efferent fibers of the amygdala are connected to some respiratory areas, such as the NTS and related areas. The breath stimulates the mechanoreceptors of the diaphragm and the visceral receptors of the organs moved during the respiratory acts, constituting a mechanism called interoception [27]. Interoception is the awareness of the body's condition based on information directly obtained from the body itself; it is also related to visceral movements when breathing in and out. The interoceptive afferent fibers are connected to autonomic and homeostatic centers in the spinal cord and in the brainstem, and then to the cingulate cortex and to the anterior dorsal posterior insula, through the thalamus-cortical tract. The interoception system can modulate the exteroceptive representation of the body, as well as the subject's tolerance of pain; the dysregulation of pathways managing or stimulating the interoception could cause a distortion of body image, affecting the subject's emotional state [27]. We can speculate that an altered function of the diaphragm may adversely affect the patient's emotional state, adversely affecting interoceptive afferents.

Pain and inflammation

The peripheral nerve structure is subjected to daily mechanical stress, as when the joint moves, it undergoes alternating phases of contraction and stretching. The correct sliding of the fascial structures of the nerve, and the nerve position between the various innervated tissues, become essential for nerve adaptation and regeneration [6]. Altered sliding may lead to dysfunction, increasing the rigidity of fascial structures during joint or respiratory movements; the nerve may develop a diameter reduction, defined as transverse contraction, and an increase in the pressure in the endoneural compartment may be observed [30]. Repetitive elongations of a nerve with reduced elasticity will further hamper nerve sliding, decreasing the blood flow, with potential ischemic changes. The fascial structures become more sensitive to mechanical stress and, after a few days of local inflammation, an action potential (similar to the mechanical stimulus that has initially caused the dysfunction) can be generated, causing inflammation at the ends of the neural pathways, such as in the spinal cord and innervated tissues, through anterograde and retrograde mechanisms. This mechanism is called ectopic electrogenesis [30]. The vagus nerve and the phrenic nerve, which innervate the diaphragm muscle, may be affected if muscular movement is limited for such a dysfunction, leading to altered sensitivity schemes and allodynia.

The vagus nerve is a mixed nerve, 20% consisting efferent cholinergic descendent fibers, and 80% afferent ascending fibers [31]. The vagus nerve, below the diaphragm, forms anastomoses with the sympathetic ganglia [24]. The vagal nerve contains large myelinated type A and B fibers, conducting efferent and visceral afferent information, and efferent preganglionic sympathetic and parasympathetic information respectively; it also includes small size, non-myelinated, visceral afferent fibers (type C), representing the largest part of the afferent compartment C [32]. The efferent fibers mainly derived from the retroambiguus nucleus (important for phrenic orders) and from the dorsal motor nucleus; the afferent fibers mainly connect to the area postrema, the spinal trigeminal nucleus, and the NTS (located in the medulla and receiving information on pain and emotions) [32]. The vagus nerve also plays an important role in the immune response and nociception. The vagus nerve perceives the inflammation and has the ability to regulate the response, influencing the hypothalamic-pituitary-adrenal gland axis, modulating the autonomic system in controlling key immune organs (spleen, adrenal glands and probably the bone marrow), and even directly through colinergic myenteric neurons [24]. This mechanism is called “cholinergic anti-inflammatory via”, acting through the inhibition of the production of pro-inflammatory cytokines, with a strategy involving the alpha7nAChR (alpha7 nicotinic acetylcholine receptor subunit).

A reduction in vagal control could stimulate inflammation, increasing the production of inflammatory cytokines [33]. It is worthy to mention that, in the population of patients with IBS, there is a dysregulation in the vagal and sympathetic system and/or a reduction in vagal tone [34]. This reduced vagal tone may be induced by mechanical stress caused by a dysfunction of the diaphragm, resulting in a compression of the nerve (with an increase in endoneural compartment pressure), which induces abnormal vagal function. In this scenario of reduced vagal control, the baroreceptors are also involved, since they are influenced by the vagus nerve and breathing. The low response of baroreceptors can increase the inflammatory response, creating a vicious circle [35]. There is a close relationship between the vagus nerve and the perception of pain. The afferents of the vagus nerve are usually able to inhibit the activity of the second order nociceptive neurons in the spinal cord, in spinothalamic and spinoreticular tracts and in the trigeminal nucleus [36].

Recent studies, however, highlight the vagus nerve’s ability to carry painful afferents to the supraspinal centers, especially with regards visceral pain [18]. This also happens for a retrograde transport of biochemical molecules through the nerve [8]. The vagus nerve also collaborates in the formation and maintenance of central pain memory, also modulating descendant inhibitory pathways connected to the nociceptive areas in the spinal cord [37]. No precise information is currently available on these ascendant (probably involving NTS, parabrachial nuclei, the periaqueductal grey area, hypothalamus, limbic area, magnum raphe, locus coeruleus) and descendant mechanisms, but we can derive that vagal tone probably has an important influence on pain perception [38]. We know that a compression of the vagus nerve can alter its function and its transport ability, just like a dysfunction of a peripheral nerve, mimicking an entrapment syndrome [39]. We can assume that abnormal tension of the diaphragm in the region of the oesophagal hiatus could cause a compression of the vagus nerve, reducing its antinociceptive and anti-inflammatory activity. The phrenic nerve originates from the spinal cord (C3-C5) and descends along the deep fascial system of the neck, reaching the mediastinum and lying on the pericardial fascia; then it crosses the diaphragm [8]. In the sub-diaphragmatic region, the phrenic nerve forms anastomoses with the celiac ganglion and the superior mesenteric ganglion and constitutes small phrenic ganglia, to finally reach the adrenal gland [24]. These connections with the sympathetic system influence the phrenic nerve with retrograde impulses from the sympathetic plexus and viscerosomatic communications. These connections are very complex and still partly unknown. The celiac and superior mesenteric ganglion are involved in the cholinergic anti-inflammatory action of the vagus nerve (to the spleen), emphasizing a parasympathetic and sympathetic action [40]. When the diaphragm muscle does not contract properly, it becomes stiff. We can assume that this could create a compression syndrome also on the phrenic nerve, altering the connections between the sympathetic and the parasympathetic system and the adrenal gland, and adversely affecting the perception of pain, the inflammatory status, and the emotional state. Based on what was previously described, this could contribute to an altered bowel function. The splanchnic nerves cross the diaphragm through small muscular spaces [8]. The sympathetic system, when activated, is able to amplify the pain and modulate the pain memory [41]. Previous studies demonstrated that the presence of vertebral osteophytes may alter the morphology of the sympathetic nerves and their function [42-43]. We can assume that if the sympathetic nerves are compressed at the level of their passage through the diaphragm, their function and morphology can change, negatively influencing the innervated tissues, including the colon.

## Conclusions

Irritable bowel syndrome (IBS) is the most commonly diagnosed gastrointestinal disorder. The patient complains of abdominal pain or discomfort and altered bowel habits in the absence of other conditions that may cause the syndrome. Patients undergo various comorbidities and a concomitant dysfunction of the diaphragm muscle. The comorbidities addressed in the article are chronic low back pain, chronic pelvic pain, chronic headache, and temporomandibular joint dysfunction, vagus nerve inflammation, and depression and anxiety; we can still find other co-morbidities, like abnormalities in upper airways and gastroesophageal reflux. Considering the neurological, fascial, and anatomical diaphragmatic connections with the lumbar area, the cervical and mandibular area, as well as its influence on the emotional sphere and on the perception of pain, we can conclude that there is a strong relationship between these co-morbidities and patients with IBS. Currently, there is no data on the pre-IBS functional status of the diaphragm muscle of these patients, as well as on their vagal and sympathetic functions. At the same time, there are no data on the potential impact of therapeutic approaches for IBS including diaphragmatic training; in the current context, we cannot quantify the influence of breathing on IBS and related pathologies. Further studies focused on the relation between breathing and IBS are needed in order to evaluate if this correlation could change therapeutic approaches to IBS.
